# Sialocele following excision of delayed-onset inflamed facial fat graft: A case report

**DOI:** 10.1097/MD.0000000000048037

**Published:** 2026-03-13

**Authors:** I Zhen Ma, Sehoon Yoon, Jae Woo Park, Ki Yong Hong

**Affiliations:** a Department of Plastic and Reconstructive Surgery, Seoul National University Hospital, Seoul National University College of Medicine, Seoul, Republic of Korea; b Department of Plastic Surgery, BIO Plastic Surgery Clinic, Seoul, Republic of Korea.

**Keywords:** autologous fat grafting, delayed inflammation, parotid gland injury

## Abstract

**Rationale:**

Autologous fat grafting is a widely used technique for facial contour restoration and is generally considered safe. Nevertheless, delayed inflammatory reactions may occur and, in rare instances, require surgical intervention. Excision of the inflamed graft in the midface region carries a potential risk of intraoperative injury to critical structures, including the facial nerve and parotid gland.

**Patient concerns:**

24-year-old woman presented with progressive swelling and tenderness of the left cheek 4 years after autologous facial fat grafting.

**Diagnosis:**

Magnetic resonance imaging revealed mixed fat necrosis and fluid collection beneath the superficial musculoaponeurotic system (sub-SMAS) plane, with extension into the masseter muscle.

**Interventions:**

As the symptoms did not improve with antibiotics and anti-inflammatory medications, surgical excision was performed. Histopathological examination revealed benign fibroadipose tissue with mild chronic inflammation and fat necrosis. Following partial excision of the inflamed graft, a postoperative sialocele developed, raising suspicion of parotid gland injury. Conservative management with anticholinergic agents and intraparotid botulinum toxin injection was initiated.

**Outcomes:**

The sialocele resolved without the need for surgical repair. At the 6-month follow-up, no recurrence was observed.

**Lessons:**

Delayed inflammation may develop after autologous fat grafting, occasionally necessitating surgical intervention. Surgical excision in the deep midface region poses a risk of intraoperative parotid injury. Awareness of this potential complication is important, and conservative management can often effectively treat parotid leakage or sialocele without further surgery in selected cases.

## 1. Introduction

Autologous fat grafting is a widely used technique to restore volume and enhance contour for both aesthetic and reconstructive purposes. However, delayed complications such as inflammation, fat necrosis, and mass formation can occur months or even years after surgery. Conservative measures such as antibiotics and anti-inflammatory medications are generally employed as a first-line management; however, persistent or progressive symptoms often necessitate surgical intervention. Surgical removal of previously grafted fat can be challenging due to altered anatomy and fibrosis at the surgical site, and may result in injury to critical structures such as the facial nerve or parotid gland.

In this article, we report a case of a patient who presented with delayed-onset inflammation in the previous graft site, which required surgical excision. Following this procedure, the patient experienced swelling at the surgical site that became more prominent with eating, raising suspicion of parotid gland injury. This symptom was successfully managed with conservative treatment.

## 2. Case presentation

According to the policies of our research institution, institutional review board approval is not required for case reports involving a single individual who cannot be identified. Therefore, institutional review board approval was waived for this case report. Written informed consent was obtained from the patient.

A 24-year-old woman presented with progressive swelling of the left cheek. The patient had previously undergone autologous fat grafting to both cheeks 4 years earlier at a local clinic for correction of a mild contour depression beneath the zygomatic arch. One year prior, she also underwent liposuction of both cheeks at another clinic. Both procedures were performed for aesthetic purposes. Swelling was initially monitored by previous physicians; however, as the symptoms persisted for more than 2 months, local corticosteroid injections were administered. This treatment led to partial improvement, but following relapse of the symptom, she was referred to our clinic for further evaluation and management.

**Figure 1.**
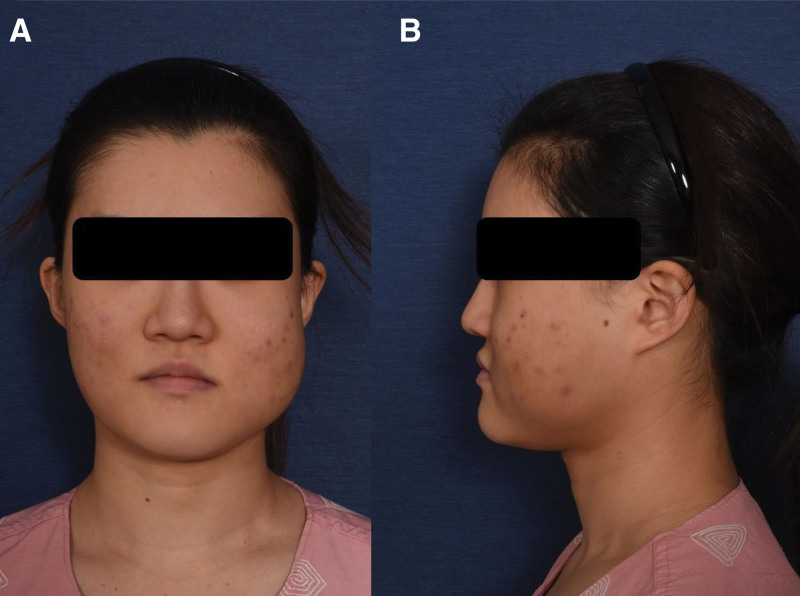
Preoperative clinical photographs. (A) Frontal view (B) Left lateral view.

**Figure 2.**
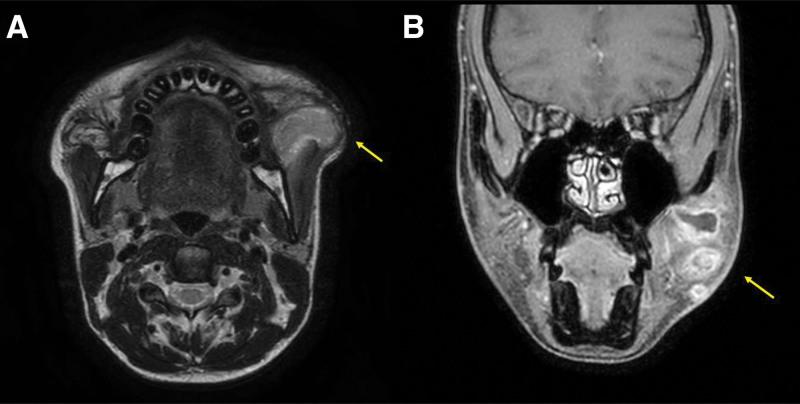
MRI revealing an infiltrative T2-hyperintense lesion (yellow arrow) beneath the SMAS (sub-SMAS) plane, with extension into the masseter muscle. (A) Axial view and (B) Coronal view. MRI = magnetic resonance imaging, SMAS = superficial musculoaponeurotic system.

On physical examination, the left cheek demonstrated swelling and mild firmness (Fig. 1). Magnetic resonance imaging (MRI) revealed an infiltrative T2-hyperintense lesion involving the masseter muscle in the left cheek, with peripheral enhancement suggestive of fat necrosis and fluid collection (Fig. 2). The lesion appeared to extend beneath the superficial musculoaponeurotic system (sub-SMAS) plane. Antibiotics and anti-inflammatory medications were administered, but without symptomatic improvement. Consequently, surgical intervention was undertaken primarily due to the progressive nature of the swelling and the diagnostic uncertainty driven by atypical MRI findings, which necessitated histopathological evaluation to definitively rule out neoplasia.

**Figure 3.**
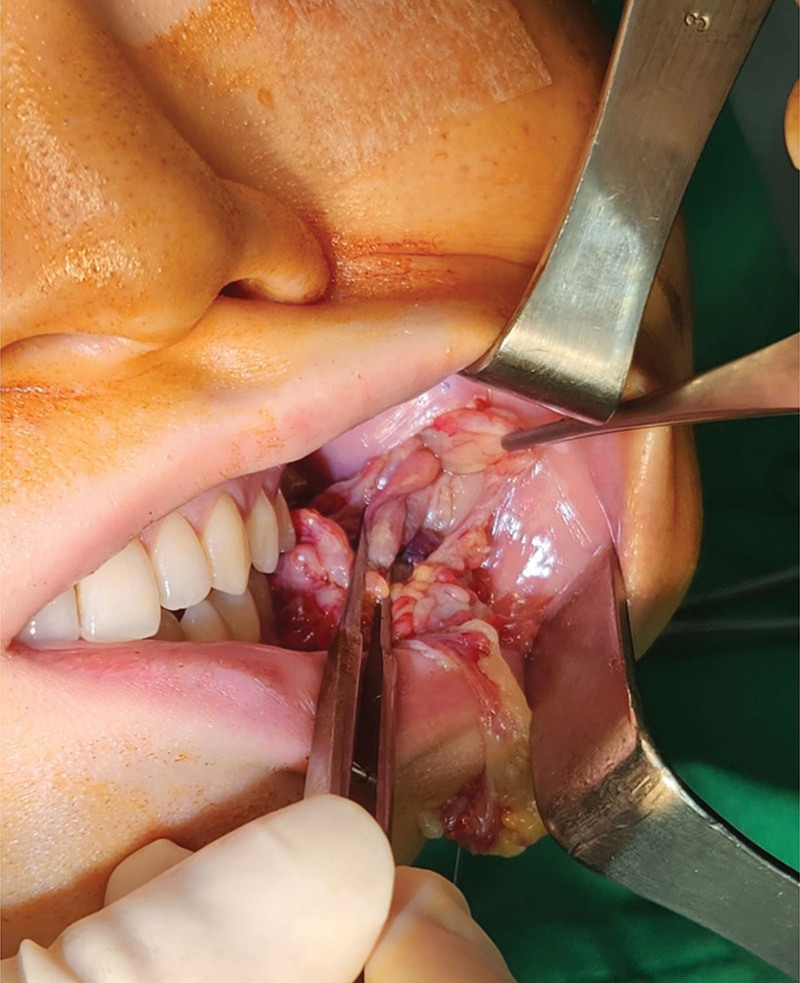
Surgical excision of the lesion performed intraorally.

**Figure 4.**
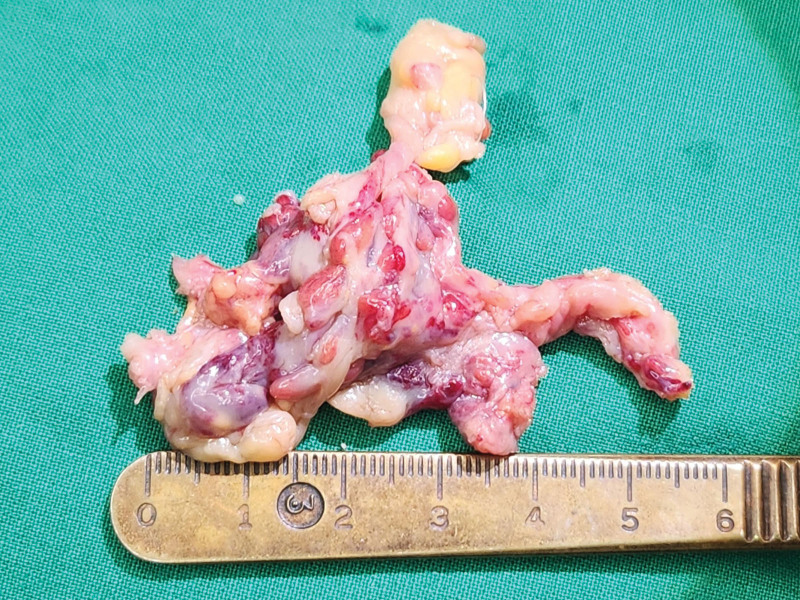
The excised specimen consisted of yellowish fatty tissue with multiple reddish nodules.

Surgical exploration and debridement of the lesion were performed. An intraoral approach was used to avoid visible scarring, and an incision was placed inferior to the Stensen duct orifice to avoid injury to the parotid duct. Dissection was performed in the sub-SMAS plane, allowing exposure of the abnormal fatty mass. The lesion was localized within the buccal fat compartment, with fibrotic adhesions to the masseter fascia, without evidence of intramuscular invasion. During the procedure, meticulous care was taken to protect the parotid duct, the buccal branches of the facial nerve, and adjacent glandular structures. The abnormal adipose tissue was then excised (Fig. 3). Grossly, the excised specimen consisted of yellowish fatty tissue with multiple reddish nodular areas (Fig. 4). Histopathologic analysis demonstrated fibroadipose tissue with vascular congestion, focal fat necrosis, and mild chronic inflammation. No parotid duct or glandular elements were identified, and there was no evidence of malignancy.

**Figure 5.**
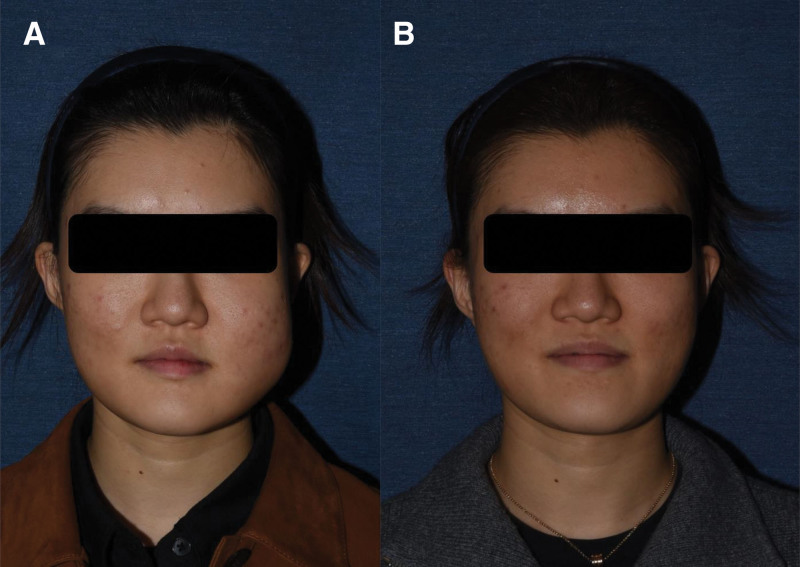
Postoperative clinical photographs. (A) One week postoperatively showing a localized sialocele in the left parotid region. (B) Six-month follow-up without recurrence.

One week postoperatively, the patient developed a localized sialocele in the left parotid region that became more pronounced with mastication, raising concern for parotid gland or duct injury (Fig. 5A). A compressive dressing was applied for 48 hours, and the collected fluid was aspirated. The patient then received an intraparotid botulinum toxin injection (12.5 IU) and was prescribed oxybutynin hydrochloride 5 mg 3 times daily for 7 days (Ditropan®; Dong Wha PHARM.CO.,LTD, Seoul, Korea). Although the sialocele recurred 2 to 3 times, repeated aspirations yielded progressively smaller volumes, and the lesion resolved within 1 month. At the 6-month follow-up, there was no evidence of recurrent swelling or discharge (Fig. 5B).

## 3. Discussion

Autologous fat graft is a widely utilized technique in facial aesthetic and reconstructive procedures due to its biocompatibility, availability, and versatility. While it is generally considered safe, delayed complications can occur, sometimes years after the initial procedure. This case highlights a rare but clinically relevant sequence of events involving delayed inflammatory response, the need for surgical excision, and subsequent salivary complications.

Delayed inflammation after autologous fat grafting is thought to result from several interrelated pathophysiologic mechanisms. A primary cause is fat necrosis, which often arises from inadequate revascularization of the grafted adipose tissue. In particular, with large-volume grafting, the central portion is at higher risk of ischemia due to insufficient revascularization, leading to adipocyte necrosis and release of intracellular lipids. Previous studies have demonstrated that a significant number of adipocytes undergo early cell death within the initial days following grafting. These necrotic adipocytes are gradually replaced by newly differentiated adipocytes derived from surviving adipose-derived stem cells or by fibrotic tissue.^[1,2]^ Small amounts of lipids may be resorbed and cleared through lymphatics, but when the volume exceeds a certain threshold, oil cysts may form, triggering a persistent sterile inflammatory response characterized by macrophage infiltration and subsequent fibrosis.^[3]^ The cumulative effect of these processes can result in granulomatous tissue formation, fibrosis, and clinical symptoms such as firmness, tenderness, or mass formation at the graft site.^[4]^

Diagnosis of delayed inflammation relies on a combination of clinical assessment, imaging studies including ultrasonography, MRI, and histopathological examination. Preoperative MRI suggested extension of the lesion into the masseter muscle; however, intraoperative findings demonstrated no true intramuscular invasion. Instead, the lesion was confined to the buccal fat compartment with fibrotic adherence to the masseteric fascia. This discrepancy was most likely due to inflammatory changes and associated edema, which can result in overestimation of the extent of tissue involvement on imaging studies. Due to the specimen’s atypical gross appearance, histopathological evaluation was performed to exclude malignancy.

While the unusually long latency of 4 years naturally raises the suspicion of an occult infection or biofilm-related process, these were considered highly unlikely. The patient exhibited no systemic signs of infection, and the lesion did not respond to empirical antibiotic therapy prior to surgery. Histopathological examination ultimately confirmed benign fibroadipose tissue with focal fat necrosis and chronic inflammation. Crucially, it demonstrated sterile inflammation without abscess formation or identifiable microorganisms, further supporting a noninfectious immune-mediated process.

These findings definitively ruled out infection and malignancy; however, they could not ascertain whether the necrotic tissue originated from the previously grafted fat or the native buccal fat. Given the patient’s history of facial fat grafting followed by buccal liposuction, the findings are most consistent with chronically remodeled grafted fat. Nevertheless, partial involvement of the native buccal fat pad cannot be entirely excluded. However, this anatomical uncertainty does not alter the clinical implications of this case. Regardless of whether the inflamed tissue originated from grafted fat or native buccal fat altered by prior liposuction, the resulting severe fibrosis and anatomical distortion pose the same surgical risks. Thus, the diagnostic approach and the need to protect the parotid gland remain broadly applicable to revisional deep midface surgeries.

Although extremely rare, isolated cases of soft tissue tumors have been reported in areas previously treated with autologous fat grafting.^[5]^ Zhao et al have hypothesized that chronic inflammation and the proliferation of mesenchymal cells in grafted adipose tissue may theoretically contribute to neoplastic transformation.^[6]^ However, the oncological risk associated with fat grafting remains controversial. Several animal and clinical studies, including those involving patients who underwent fat grafting after breast-conserving surgery, have not shown an increased risk of local recurrence or tumorigenesis.^[7–9]^

Initial management of delayed inflammation typically involves conservative measures such as anti-inflammatory medications, corticosteroid injections, or aspiration. However, these approaches are often insufficient for definitive resolution. Inflammatory nodules or granulomas may recur after aspiration, and corticosteroids may offer only temporary relief without eliminating the underlying necrotic or infected tissue. When necrotic adipose tissue becomes encapsulated, immune clearance is impaired, allowing inflammation to persist. This may lead to the formation of granulomas, which can cause recurrent swelling and discomfort. In such cases, surgical excision may be considered to alleviate symptoms and prevent further inflammatory episodes that can negatively affect the patient’s quality of life. In our patient, the primary indications for surgical excision were the failure of conservative management and the need to resolve diagnostic uncertainty to exclude malignancy.

In a retrospective cohort study of patients who underwent mastectomy and fat grafting, fat necrosis developed in 10.5% of cases, and approximately 5% required biopsy or surgical excision due to persistent nodular masses with diagnostic uncertainty.^[10]^ Jang et al described the limited efficacy of conservative management in granulomatous reactions caused by various injection materials, emphasizing the role of surgical intervention.^[11]^ Moreover, excision enables histopathological evaluation to exclude rare but serious conditions such as malignancy or atypical infection. Therefore, timely surgical removal not only provides symptom relief and aesthetic restoration but also contributes to diagnostic certainty and improved long-term outcomes.

Surgical removal of previously grafted fat, particularly in the midface region, carries a risk of injury to adjacent anatomical structures. The parotid duct runs over the superficial surface of the masseter, then turns medially to pierce the buccinator at its anterior border and opens into the oral vestibule opposite the maxillary second molar. The buccal and zygomatic branches of the facial nerve, which course superficially across the masseter muscle and beneath the SMAS are vulnerable to injury during deep cheek dissection. Distorted tissue planes, dense fibrosis, and limited visibility in revisional surgeries further increase the risk of iatrogenic injury to adjacent tissues. This underscores the importance of anatomical awareness, meticulous surgical technique, and appropriate diagnostic consideration when operating near the parotid duct and glandular structures.

Salivary fistulas or leakage following parotid gland injury can usually be managed conservatively, especially when the injury is partial or small. First-line treatments include anticholinergic medications (such as glycopyrrolate or scopolamine) to reduce salivary output, compressive dressings to promote ductal healing, and dietary modifications to minimize salivary stimulation.^[12]^ In recent years, botulinum toxin type A has emerged as an effective and minimally invasive modality for the treatment of salivary leakage or fistulas. Botulinum toxin acts by inhibiting acetylcholine release at the parasympathetic nerve terminals, thereby reducing salivary gland secretion.^[13,14]^ In most cases, conservative management is sufficient for resolution within 2 to 4 weeks. However, if symptoms persist or recur despite initial treatment, additional interventions may be necessary. These include repeated needle aspiration and placement of suction drains, which help reduce fluid accumulation and promote fistula closure. Sclerotherapy using hot hypertonic saline can be employed to induce localized fibrosis and closure of the fistula within a few days. If conservative options fail, surgical interventions such as ductal anastomosis or ductal ligation may be required.^[12,15]^

In our case, the lesion was located in the left cheek near the buccal fat pad, placing the parotid duct and glandular structures at risk during excision. The development of the sialocele is best understood as a multifactorial complication rather than a simple iatrogenic injury. Severe fibrosis from prior fat grafting and liposuction distorted the local tissue planes, tethering the inflammatory lesion to the parotid fascia. While histology showed no ductal tissue, the clinical presentation indicates a superficial parenchymal disruption, which likely occurred as unavoidable micro-trauma while dissecting these highly adherent structures. Although diagnostic tests such as dye leakage testing, saline injection, or sialography can aid in differentiating between ductal and parenchymal injuries, they were not because diagnosis can typically be done by clinical presentation and history taking.^[16]^

When excision of localized mass in deep parotid region is planned, choosing an external approach can significantly increase the risk of facial nerve injury. Among iatrogenic postoperative complications, a sialocele generally has a more favorable prognosis than facial nerve injury because it typically resolves spontaneously with minimal sequelae. Accordingly, the intraoral approach used in this case was a safer and more reasonable route for access and removal than an external approach. In addition, the intraoral approach avoids a visible facial scar. Our patient unfortunately developed sialocele, which led to anticholinergic medication in combination with a botulinum toxin injection into the parotid gland. Sialocele gradually resolved within 1 month, without the need for further surgical intervention. Although no confirmatory imaging such as sialography was performed to evaluate for residual fistula or sialocele, there were no clinical signs of recurrence after 6-month follow-up. An asymptomatic course over 6 months is generally considered as complete healing, and no further imaging study was done. Ultimately, the patient’s initial symptoms, such as swelling and firmness, resolved completely after excision. Notably, no contour depression or volume deficiency was noted postoperatively, and the patient expressed satisfaction with the cosmetic outcome.

This case report has several limitations. First, there was insufficient information about the patient’s condition during the 4 years after the initial fat grafting, limiting our ability to define the pathogenesis of the atypical fatty mass. Second, it is unclear whether the liposuction procedure affected the fascial structure of the buccal fat compartment. Any damage to this layer could have altered tissue anatomy or contributed to abnormal fat displacement or remodeling. Third, the volume, location, and method of initial fat injection were not recorded, which makes it difficult to determine whether the lesion originated from the grafted fat or from displacement and degeneration of buccal fat. A similar case has been reported after facelift surgery, where injury to the buccal fat fascia resulted in pseudoherniation of the fat pad as a rounded cheek mass.^[17]^ While this mechanism cannot be confirmed in this case, it may be relevant and should be considered.

This case underscores the importance of recognizing delayed-onset inflammatory complications following autologous facial fat grafting. Complications such as fat necrosis, oil cyst formation, and chronic inflammation may not always respond adequately to conservative management. When symptoms persist despite nonsurgical treatment, surgical excision may be considered for definitive resolution and diagnostic clarity in selected cases. While the intraoral approach may reduce the risk of facial nerve injury compared with an external approach, it carries a potential risk of parotid gland injury. If salivary leakage occurs, this case suggests that conservative management with anticholinergic agents or botulinum toxin can effectively resolve the complication without further surgery.

## Author contributions

**Conceptualization:** Ki Yong Hong.

**Data curation:** Jae Woo Park.

**Investigation:** Jae Woo Park, Ki Yong Hong.

**Supervision:** Ki Yong Hong.

**Writing – original draft:** I Zhen Ma, Sehoon Yoon.

**Writing – review & editing:** I Zhen Ma, Sehoon Yoon.
